# Real-Time Monitoring of Strain Accumulation and Relief during Epitaxy of Ultrathin Co Ferrite Films with Varied Co Content

**DOI:** 10.3390/ma16237287

**Published:** 2023-11-23

**Authors:** Jannis Thien, Jari Rodewald, Tobias Pohlmann, Kevin Ruwisch, Florian Bertram, Karsten Küpper, Joachim Wollschläger

**Affiliations:** 1 Department of Physics, Osnabrück University, 49076 Osnabrück, Germany; jthien@uos.de (J.T.); jari.rodewald@gmx.de (J.R.); pohlmann_t@t-online.de (T.P.); kruwisch@uos.de (K.R.); kkuepper@uos.de (K.K.); 2 Deutsches Elektronen-Synchrotron (DESY), Photon Science, 22607 Hamburg, Germany; florian.bertram@desy.de

**Keywords:** cobalt ferrite, ultrathin films, strain, X-ray diffraction

## Abstract

Ultrathin Co${}_{x}$Fe${}_{3-x}$O${}_{4}$ films of high structural quality and with different Co content (*x* = 0.6–1.2) were prepared by reactive molecular beam epitaxy on MgO(001) substrates. Epitaxy of these ferrite films is extensively monitored by means of time-resolved (operando) X-ray diffraction recorded in out-of-plane geometry to characterize the temporal evolution of the film structure. The Co ferrite films show high crystalline ordering and smooth film interfaces independent of their Co content. All Co${}_{x}$Fe${}_{3-x}$O${}_{4}$ films exhibit enhanced compressive out-of-plane strain during the early stages of growth, which partly releases with increasing film thickness. When the Co content of the ferrite films increases, the vertical-layer distances increase, accompanied by slightly increasing film roughnesses. The latter result is supported by surface-sensitive low-energy electron diffraction as well as X-ray reflectivity measurements on the final films. In contrast, the substrate–film interface roughness decreases with increasing Co content, which is confirmed with X-ray reflectivity measurements. In addition, the composition and electronic structure of the ferrite films is characterized by means of hard X-ray photoelectron spectroscopy performed after film growth. The experiments reveal the expected increasing ${\mathrm{Fe}}^{3+}$/${\mathrm{Fe}}^{2+}$ cation ratios for a higher Co content.

## 1. Introduction

Among transition metal (TM) ferrites, the ferrimagnetic semiconductor CoFe${}_{2}$O${}_{4}$ (CFO) is a key material in the field of spintronics. For ultrathin epitaxial ferrite films, the strain induced by the lattice mismatch between the ferrite lattice and the substrate lattice is quite capable of leaving a great impact on several physical and chemical properties of ferrite films [1,2,3,4,5]. In particular, it has been reported that the strain in thinner ferrite films can modify the cationic distribution on different lattice sites [6], which in turn significantly affects, e.g., the electronic and magnetic properties of the ferrite films [1,7,8]. Therefore, substrate-induced strain can be specifically used for strain-engineering of ultrathin ferrite films to hugely improve the performance of ferrites for spintronic applications such as, e.g., spin-filters, where highly spin-polarized electrons are generated due to spin-dependent tunneling through ferrimagnetic barriers [9,10,11,12]. However, in order to tailor the properties of these ultrathin ferrite films using strain, it is important to know the details of strain accumulation, especially during the very early stages of growth, as well as strain relief during later growth stages [13]. Moreover, interfaces formed during the early stages of film growth are also crucial for the quality of devices based on, e.g., spin Hall magnetoresistance [14].

Hence, this work focuses on time-resolved (operando) X-ray diffraction (XRD) [13,15] to study the growth behavior and evolving strain of Co${}_{x}$Fe${}_{3-x}$O${}_{4}$ thin films with varying Co content *x* = 0.6–1.2 up to a final film thickness of about 30 nm. The films are prepared using reactive molecular beam epitaxy (RMBE) with two individual sources for Fe and Co while deposition is performed in a diluted oxygen atmosphere to oxidize directly the deposited metals during deposition and to form Co ferrite films. The Co${}_{x}$Fe${}_{3-x}$O${}_{4}$ films were grown on MgO(001) substrates (lattice constant $a_{\mathrm{MgO}}$ = 421.2 pm) motivated by the small lattice mismatch of about −0.33% (comparing two unit cells of MgO with a single unit cell of CFO, lattice constant $a_{\mathrm{CFO}}$ = 839.2 pm). The latter agrees very well with the lattice constant $a_{{\mathrm{Fe}}_{3}O_{4}}$ = 839.6 pm of magnetite (Fe${}_{3}$O${}_{4}$ being Co${}_{x}$Fe${}_{3-x}$O${}_{4}$ with x=0), which has been studied here too for reasons of comparison. Thus, independent of the Co content of the Co ferrite films, almost-perfect growth conditions are guaranteed for Co${}_{x}$Fe${}_{3-x}$O${}_{4}$ thin films on MgO(001) due to the small lattice mismatch obtained for both Fe${}_{3}$O${}_{4}$ and CoFe${}_{2}$O${}_{4}$.

In addition to XRD, post-deposition structural characterization is performed by means of X-ray reflection (XRR) and low-energy electron diffraction (LEED) as well as electronic and magnetic characterization by means of hard X-ray photoelectron spectroscopy (HAXPES) to shed light on the surface and interface properties as well as on the electronic properties of the Co ferrite films and to obtain a more conclusive and comprehensive analysis.

## 2. Materials and Methods

Both the preparation and the in situ characterization of the Co${}_{x}$Fe${}_{3-x}$O${}_{4}$ films were carried out in an ultra-high-vacuum chamber with a base pressure of $1\times 10^{-10}$ mbar at beamline I07 [16] of the diamond light source (DLS). Prior to film deposition, the MgO(001) substrates were cleaned at a temperature of 400 °C in $5\times 10^{-5}$ mbar O${}_{2}$ atmosphere to remove contaminating adsorbates (e.g., carbon) from the surfaces and to obtain well-defined surfaces [17]. The effectiveness of the cleaning process was examined with LEED.

Co${}_{x}$Fe${}_{3-x}$O${}_{4}$ films were grown by evaporating metals from pure Co and Fe rods in $5\times 10^{-6}$ mbar O${}_{2}$ atmosphere, while keeping the substrates at a temperature of 250 °C. The metal rods (targets) were heated by exposing them directly to bombardment with electrons of high energy. It was demonstrated previously that ultrathin Fe${}_{3}$O${}_{4}$ films of high quality are formed on MgO(001) under these growth conditions [18]. In order to steer the stoichiometry of the resulting Co${}_{x}$Fe${}_{3-x}$O${}_{4}$ films, the Co flux was varied, whereas the Fe flux was kept constant. Additionally, one Fe${}_{3}$O${}_{4}$ film was grown under the same conditions for reasons of comparison.

During film growth, XRD measurements were continuously carried out in specular (θ–2θ) out-of-plane geometry close to the (002) and (004) Bragg conditions of MgO and Co${}_{x}$Fe${}_{3-x}$O${}_{4}$, respectively, to monitor the growth behavior of the ferrite films of different stoichiometry. Immediately after film deposition, LEED measurements and XRR measurements were performed at room temperature to examine the surface structure and crystallinity of the prepared Co${}_{x}$Fe${}_{3-x}$O${}_{4}$ film surfaces and to determine their final film thicknesses, respectively. Post-deposition XRD was also performed to examine a larger range of q-space in more detail not available in tr-XRD during deposition of the Co ferrite films. For the XRD and XRR measurements, a photon energy of 21 keV and a two-dimensional Pilatus 100K detector (Dectris AG, Baden, Switzerland) was used.

After film growth, HAXPES experiments were conducted at beamline P22 of PETRA III at Deutsches Elektronen-Synchrotron (DESY) (Hamburg, Germany) [19] and at 7-ID of the National Synchrotron Light Source II (NSLS-II) (Upton, NY, USA) [20] to examine the electronic structure and to determine the chemical composition of the Co${}_{x}$Fe${}_{3-x}$O${}_{4}$ films. Here, Co 2p, Fe 2p, and O 1s photoelectrons were excited by 6 keV photons in both studies.

The stoichiometry *x* of each film was determined by evaluating the relative intensity ratios of the Co 2p and Fe 2p spectra. The intensities were corrected by subtracting a Shirley background and normalized to the corresponding photoionization cross sections from Scofield [21] as well as to the corresponding inelastic mean free paths calculated by the Tanuma, Powell, and Penn formula (TPP-2M) [22]. In addition, the O content was obtained analogously by comparing the normalized intensities of O 1s photoelectrons with intensities obtained from Co and Fe cations. For all films, the obtained O stoichiometries match with the expected O stoichiometry in Co${}_{x}$Fe${}_{3-x}$O${}_{4}$ (cation:anion = 4:3), indicating negligible anionic and/or cationic defects.

## 3. Results and Discussion

### 3.1. Operando tr-XRD during Film Growth

**Figure 1:**
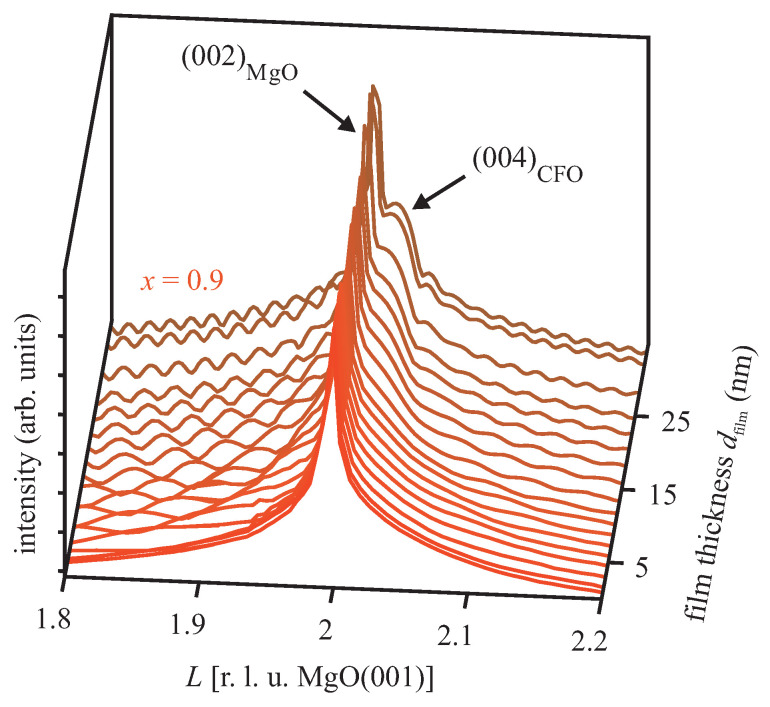
XRD scans in specular (θ–2θ) geometry close to the (002) Bragg peak of MgO. The (004) Bragg peak of the Co${}_{0.9}$Fe${}_{2.1}$O${}_{4}$ film evolves during film deposition. Clear Laue oscillations are visible due to homogeneous film thickness and smooth interfaces.

During deposition of the ferrite films, the XRD diffractograms were recorded in out-of-plane geometry to examine both the (002) Bragg reflection of the MgO substrate as well as the (004) Bragg peak of the evolving Co ferrite film. For operando XRD studies during growth of the Co ferrite films, Figure 1 exemplifies this experiment, showing XRD scans along the (00L) rods for different times during film growth of Co${}_{0.9}$Fe${}_{2.1}$O${}_{4}$.

Initially, the XRD scan of the pristine MgO(001) substrate shows exclusively a sharp and intense Bragg peak located at L=2 corresponding to the (002) Bragg reflection of MgO with a rock-salt structure. Here *L* denotes the (vertical) scattering vector *q* scaled to the Bragg conditions of the MgO(001) substrate, namely, $L=qa_{\mathrm{MgO}}/2\pi$. After the first few monolayers of Co${}_{0.9}$Fe${}_{2.1}$O${}_{4}$ (∼2 nm) are deposited on the MgO substrate, an initially very broad peak at slightly larger *L* values than the MgO(002) Bragg reflection becomes apparent as a shoulder. This can be ascribed to the evolving (004) Bragg reflection of Co${}_{0.9}$Fe${}_{2.1}$O${}_{4}$ due to its (inverse) spinel structure and vertical-layer distance $c_{\mathrm{vert}}$ smaller than the MgO layer distance. With increasing coverage, the (004) Bragg reflection of Co${}_{0.9}$Fe${}_{2.1}$O${}_{4}$ gains intensity and its FWHM decreases due to the growing thickness of the ferrite films. Moreover, Laue fringes emerge, pointing to the formation of high-quality ferrite films (homogeneous film thickness, high crystalline order).

**Figure 2:**
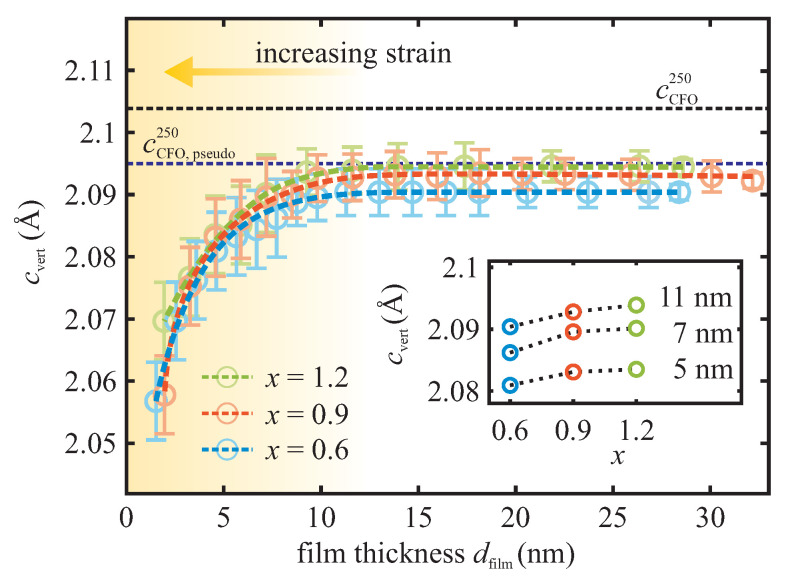
Temporal evolution of the vertical-layer distance $c_{\mathrm{vert}}$ during growth of Co${}_{x}$Fe${}_{3-x}$O${}_{4}$ films. The corresponding dashed lines serve as guides to the eye. The vertical-layer distance $c_{\mathrm{CFO}}^{250}$ of bulk CFO at 250 °C and the vertical-layer distance $c_{\mathrm{CFO},\mathrm{pseudo}}^{250}$ of pseudomorphic CFO films grown at 250 °C are displayed for reference (dashed horizontal lines). Representative at three different Co${}_{x}$Fe${}_{3-x}$O${}_{4}$ film thicknesses, the inset shows the dependence of the evolving layer distances $c_{\mathrm{vert}}$ of the Co${}_{x}$Fe${}_{3-x}$O${}_{4}$ films on the Co content.

Each XRD scan was analyzed in the framework of kinematical diffraction theory to determine the vertical-layer distance $c_{\mathrm{vert}}$. The temporal evolution of the vertical-layer distance $c_{\mathrm{vert}}$ as obtained from this analysis is depicted in Figure 2 for Co${}_{x}$Fe${}_{3-x}$O${}_{4}$ films with varied Co content *x*.

The temporal evolution of the vertical-layer distances $c_{\mathrm{vert}}$ of the evolving film presented in Figure 2 shows that they are smaller than the layer distance $c_{\mathrm{CFO}}^{250}$ = 210.3 nm of bulk CFO at 250 °C for all Co${}_{x}$Fe${}_{3-x}$O${}_{4}$ films during the entire growth process. Here, thermal expansion of both the ferrite film and the MgO substrate has been considered to calculate the lattice constants at 250 °C from room temperature values [23]. Thus, the Co${}_{x}$Fe${}_{3-x}$O${}_{4}$ films are vertically compressively strained from the very first growth stages. Generally, this kind of compressive strain in the vertical direction is caused by lateral tensile strain, which can be expected, e.g., considering lateral adaptation of the Co${}_{x}$Fe${}_{3-x}$O${}_{4}$ unit cell to the slightly larger unit cell of MgO(001) (pseudomorphic growth).

In general, the vertical induced strain $\Delta c_{\mathrm{vert}}/c_{\mathrm{vert}}$ caused by the lateral elastic distortion $\Delta a_{\mathrm{lat}}$ of the Co${}_{x}$Fe${}_{3-x}$O${}_{4}$ unit cells can be determined quantitatively according to Hashimoto et al. [24] via

(1) $$\frac{\Delta c_{\mathrm{vert}}}{c_{\mathrm{vert}}}=\frac{2\nu }{\nu -1}\frac{\Delta a_{\mathrm{lat}}}{a_{\mathrm{lat}}}$$

with the in-plane film lattice constant $a_{\mathrm{lat}}$ and ν=0.367 as the Poisson ration of CFO [25]. Assuming indeed a complete lateral adaptation of the Co${}_{x}$Fe${}_{3-x}$O${}_{4}$ lattice to the MgO(001) lattice and taking also into account the thermal expansion of both materials [23,26], a lateral strain of $\Delta a_{\mathrm{lat}}/a_{\mathrm{CFO}}^{250}$ = 0.35% is expected, resulting in a vertical compressive strain of $\Delta c_{\mathrm{vert}}/c_{\mathrm{CFO}}^{250}$ = −0.41% (cf. Figure 2).

The expected vertical compression of the films expected for pseudomorphic growth of CFO on MgO(001) is observed during the late stages of growth (film thickness $d_{\mathrm{film}}>$ 12 nm). For the early growth stages ($d_{\mathrm{film}}<$ 12 nm), however, the Co${}_{x}$Fe${}_{3-x}$O${}_{4}$ films show a significantly larger vertical compressive strain than expected and, thus, point to a large tensile lateral strain. Quite similar behavior has recently been observed in ultrathin Ni${}_{x}$Fe${}_{3-x}$O${}_{4}$ films (0≤x≤1.5) [13]. This effect has been attributed to the formation of antiphase boundaries (APBs) caused by the different crystal structure of the film (inverse spinel) and substrate (rock salt). It has been reported that the APBs significantly influence the strain properties of films [27,28]. APBs in the films lead to an increased lateral expansion and, consequently, to additional compressive vertical strain (cf. Equation (1)). With increasing film coverage, the compressive out-of-plane strain in each film diminishes constantly as the APB density decreases [29,30] until, finally, only a constant residual strain of −0.5(±0.1)% remains at >12 nm film thickness. This residual strain is in accordance with the expected vertical strain of −0.41% assuming pseudomorphic growth (see above).

Due to the small lattice mismatch, the incorporation of strain-releasing misfit dislocations in the films would not be presumed to occur up to a critical film thickness. According to the model of Matthews and Blakeslee [31], the critical film thickness is ∼87 nm for CFO films deposited on MgO(001). Thus, partial relaxation of the CFO films due to the injection of misfit dislocations is not expected here. Furthermore, we would like to point out that vertical compressive strain is decreasing with increasing Co content *x* in the Co${}_{x}$Fe${}_{3-x}$O${}_{4}$ films (cf. inset of Figure 2).

### 3.2. Post-Deposition Characterization

After film deposition, several experimental techniques were used to characterize the Co ferrite films in more detail. LEED, XRR and XRD experiments were performed in situ. Further HAXPES studies were carried out after the transfer of the samples through ambient conditions.

#### 3.2.1. LEED

**Figure 3:**
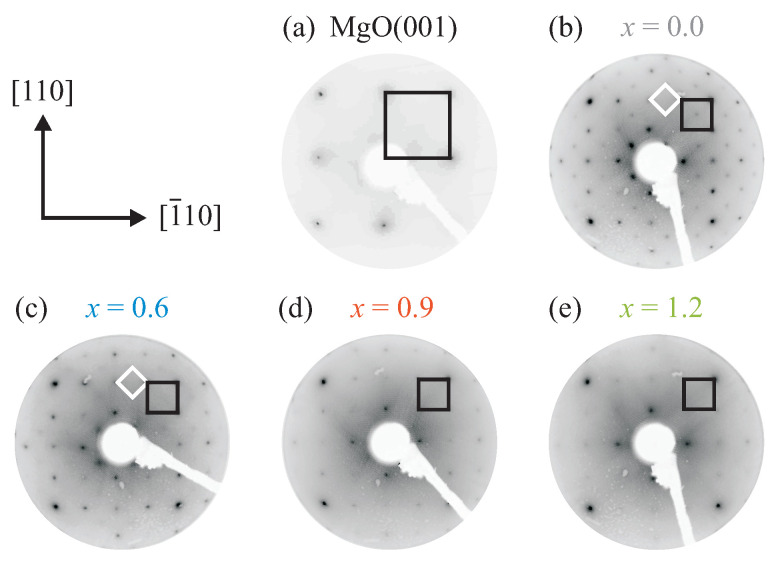
LEED pattern of the cleaned MgO(001) substrate and Co${}_{x}$Fe${}_{3-x}$O${}_{4}$ films recorded at an electron energy of 150 eV. The black squares indicate (1×1) surface unit cells of substrate and film. The white squares indicate the $\left(\sqrt{2}\times \sqrt{2}\right)R45^{{}^{\circ}}$ superstructure unit cells.

LEED images recorded at 150 eV electron energy directly after Co${}_{x}$Fe${}_{3-x}$O${}_{4}$ film growth are displayed in Figure 3. A typical LEED image of the pristine MgO(001) substrate after the cleaning procedure is displayed as well.

The diffraction pattern of the cleaned MgO(001) substrate features a clear square (1×1) surface unit cell (reciprocal unit vectors point in the [110] and $\left[\bar{1}10\right]$ directions) with sharp reflections and a low background intensity, indicating a well-ordered and crystalline MgO(001) surface with a low defect density.

For the Co${}_{x}$Fe${}_{3-x}$O${}_{4}$ film with x=0 (magnetite, Fe${}_{3}$O${}_{4}$), the LEED pattern also shows a clear square fundamental (1×1) surface unit cell with Fe${}_{3}$O${}_{4}\langle 110\rangle ∥$ MgO〈110〉. The reciprocal surface unit cell of Fe${}_{3}$O${}_{4}$ is approximately two times smaller than the surface unit cell of MgO(001) in both crystrallographic directions due to the almost doubled size of the surface unit cell of Fe${}_{3}$O${}_{4}$(001) compared to the unit cell of MgO(001). Additionally, a $\left(\sqrt{2}\times \sqrt{2}\right)R45^{{}^{\circ}}$ superstructure is visible, which is typical for well-ordered Fe${}_{3}$O${}_{4}$ surfaces [32,33,34].

As the Co content *x* in the Co${}_{x}$Fe${}_{3-x}$O${}_{4}$ films increases, the intensity of the $\left(\sqrt{2}\times \sqrt{2}\right)R45^{{}^{\circ}}$ superstructure diffraction pattern decreases until it vanishes completely for x>0.6. The diffraction pattern of the fundamental (1×1) structure, however, shows intense and sharp diffraction peaks. This result is in excellent agreement with earlier studies demonstrating that the surface of CoFe${}_{2}$O${}_{4}$(001) films is not reconstructed [35]. Furthermore, a marginal increase in the background intensity with increasing Co content is observable, pointing to a slightly increasing density of surface defects for the Co${}_{x}$Fe${}_{3-x}$O${}_{4}$ films with a higher Co content.

#### 3.2.2. XRR

**Figure 4:**
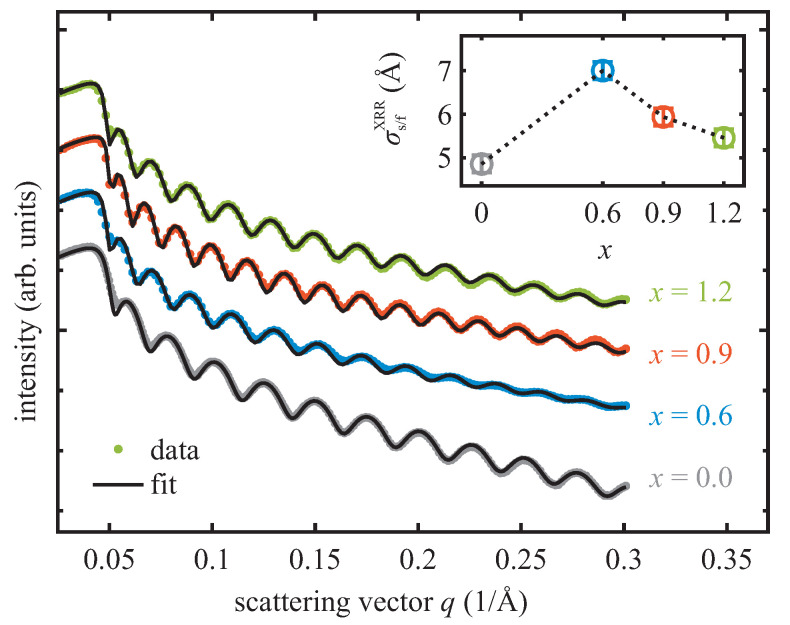
Reflectivity curves and respective fits (black solid lines) for varied Co${}_{x}$Fe${}_{3-x}$O${}_{4}$ films with different Co content (0≤x≤1.2). The inset shows the interface roughness $\sigma_{s/f}^{\mathrm{XRR}}$ between MgO substrate and each Co${}_{x}$Fe${}_{3-x}$O${}_{4}$ film extracted from the respective fits.

The X-ray reflectivity scans of all Co${}_{x}$Fe${}_{3-x}$O${}_{4}$ films recorded directly after film deposition are depicted in Figure 4. All reflectivity curves exhibit clear intensity oscillations (Kiessig fringes) due to interference of the beams reflected from the smooth film surface and substrate–film interfaces pointing to sharp interfaces. In each reflectivity curve, the intensity oscillations show only one periodicity, indicating the formation of single Co${}_{x}$Fe${}_{3-x}$O${}_{4}$ films of 30(±2) nm thickness (cf. Table 1 for more details). The oscillations are most strongly attenuated for the Co${}_{x}$Fe${}_{3-x}$O${}_{4}$ film with the lowest Co content of *x* = 0.6. The Kiessig fringes are more prominent for the pure magnetite film and for Co ferrite films with a higher Co content. Thus, it can be concluded that the interface roughness decreases with increasing Co content.

The reflectivity curves were analyzed according to the Parratt algorithm [36] and the Névot–Croce roughness model [37]. For comparison, the corresponding calculated reflectivity curves (black lines) are shown in Figure 4 as well. The fits are based on a single-film-plus-substrate model, in which the film thickness, interface roughness and refractive indices are fitting parameters. For the refractive index of the MgO substrate, literature values [38] were used, whereas for the refractive indices of the Co${}_{x}$Fe${}_{3-x}$O${}_{4}$ films a deviation of ±5% from the reported value for bulk CFO [38] was allowed. The calculated reflectivity curves agree well with the experimental data for all samples. This confirms the applied model and the formation of single Co${}_{x}$Fe${}_{3-x}$O${}_{4}$ films. The dependence of the interface roughness $\sigma_{s/f}^{\mathrm{XRR}}$ on different Co content is presented in Figure 4. It has its maximum for *x* = 0.6, while it decreases with increasing Co content as already discussed qualitatively before considering the attenuation of the Kiessig fringes.

#### 3.2.3. Post-Deposition XRD

**Figure 5:**
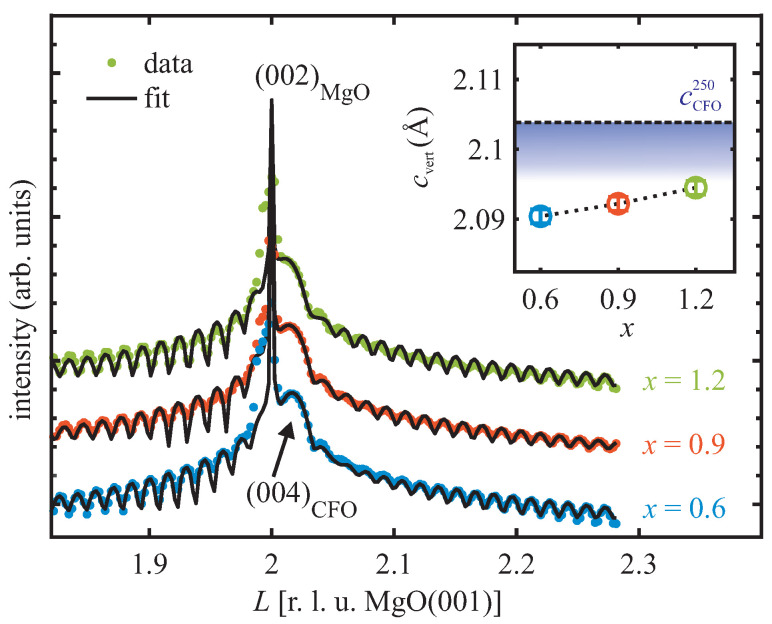
XRD scans (dots) of Co${}_{x}$Fe${}_{3-x}$O${}_{4}$ film (x≥0.6) recorded after deposition at 250 °C. Corresponding diffractograms calculated using kinematic diffraction theory (black solid lines) are also shown. The obtained vertical-layer distances $c_{\mathrm{vert}}$ are presented in the inset. The horizontal dashed line in the inset marks the vertical-layer distance $c_{\mathrm{CFO}}^{250}$ of bulk CFO at 250 °C considering thermal expansion. All films are vertically compressed with decreasing compression for increasing Co content.

Figure 5 presents the experimental XRD data of different Co${}_{x}$Fe${}_{3-x}$O${}_{4}$ films recorded directly after film growth. The (004) Bragg reflections of the Co${}_{x}$Fe${}_{3-x}$O${}_{4}$ films shift closer to the (002) Bragg reflection of MgO as the Co content increases, indicating a continuously increasing vertical-layer distance with increasing Co content (cf. inset of Figure 5). All films are nearly atomically flat, as demonstrated by the well-developed Laue fringes.

For quantitative analysis, XRD diffractograms were additionally calculated via kinematic diffraction theory, also shown as black lines in Figure 5. They were optimized while varying the structural parameters such as, e.g., the vertical-layer distance $c_{\mathrm{vert}}$ as well as the roughness $\sigma_{f}^{\mathrm{XRD}}$ and $\sigma_{s/f}^{\mathrm{XRD}}$ of the film surface and substrate–film interface, respectively. The calculated diffractograms agree well with the experimental data. The respective structural parameters are presented in Table 1. In accordance with the LEED analysis, the surface roughness $\sigma_{f}^{\mathrm{XRD}}$ is very small and the interface roughness $\sigma_{s/f}^{\mathrm{XRD}}$ obtained using XRD is in excellent agreement with those concluded from XRR (cf. Figure 4).

### 3.3. HAXPES

**Figure 6:**
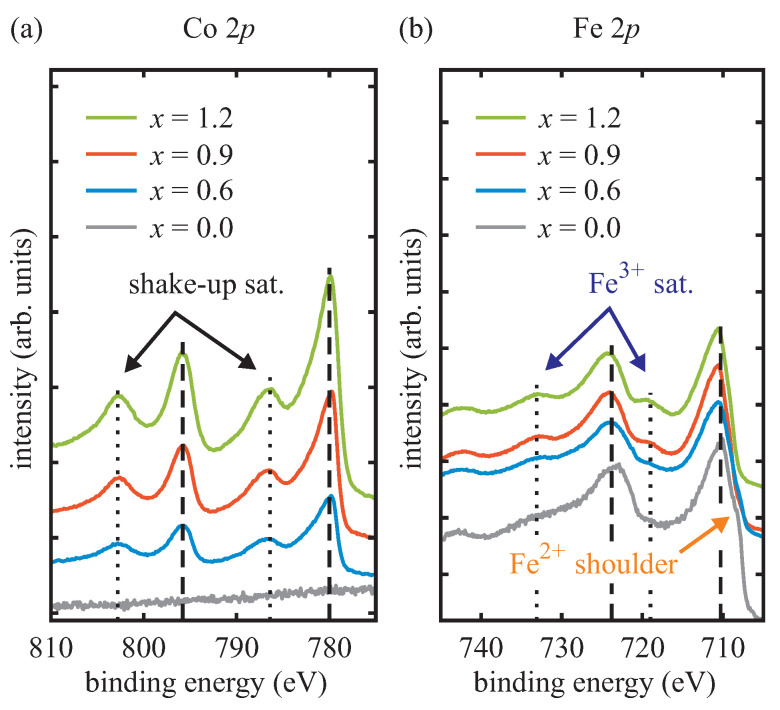
(**a**) Co 2p and (**b**) Fe 2p core-level spectra of Co${}_{x}$Fe${}_{3-x}$O${}_{4}$ films with different Co content. Dotted black lines indicate the positions of ${\mathrm{Co}}^{2+}$ shake-up satellites in (**a**) and the positions of the ${\mathrm{Fe}}^{3+}$ charge-transfer satellite peaks in (**b**). Dashed black lines indicate the positions of the respective main peaks for CFO (x=1) in (**a**) and for magnetite (x=0) in (**b**).

The Co 2p and Fe 2p core-level spectra of the HAXPES measurements of previously prepared Co${}_{x}$Fe${}_{3-x}$O${}_{4}$ films recorded after deposition and sample transfer under ambient conditions are shown in Figure 6. The spectra were calibrated according to the O 1s core-level at 530 eV [39,40]. All spectra show $2p_{1/2}$ and $2p_{3/2}$ photoelectron peaks as a result of spin-orbit coupling.

All Co 2p core-level spectra of the Co${}_{x}$Fe${}_{3-x}$O${}_{4}$ films with x≥0.6 show two main peaks (Co $2p_{1/2}$ and Co $2p_{3/2}$), which are located at binding energies of about 795.8 eV and 780.0 eV, respectively. As expected, there is no Co 2p signal for the Fe${}_{3}$O${}_{4}$ film (x=0, cf. gray line in Figure 6a). Each main peak is accompanied by one (shake-up) satellite peak at ∼6 eV higher binding energies characteristic for divalent Co cations [35,41,42]. The shape of the presented Co 2p spectra is therefore consistent with ${\mathrm{Co}}^{2+}$ incorporated in ferrite films [35].

The Fe 2p core-level spectrum of the magnetite film (x=0) reveals two main peaks of Fe $2p_{1/2}$ and Fe $2p_{3/2}$ at binding energies of about 723.8 eV and 710.3 eV, respectively. The Fe $2p_{3/2}$ peak also shows a shoulder at its lower-binding-energy side due to the presence of ${\mathrm{Fe}}^{2+}$ cations [43]. Furthermore, no *apparent* (charge-transfer) satellite can be observed between the Fe $2p_{1/2}$ and Fe $2p_{3/2}$ peaks. This effect is attributed to mixed ${\mathrm{Fe}}^{3+}$ and ${\mathrm{Fe}}^{2+}$ valence states, known well from magnetite [39].

As the Co content in the Co${}_{x}$Fe${}_{3-x}$O${}_{4}$ films increases (x>0), both the Fe $2p_{1/2}$ peak and Fe $2p_{3/2}$ peak shift to higher binding energies. In addition, the ${\mathrm{Fe}}^{2+}$-related shoulder vanishes for higher Co content while ${\mathrm{Fe}}^{3+}$ charge-transfer satellites at ∼719.0 eV and ∼733.0 eV become visible. All three observations point to increasing ${\mathrm{Fe}}^{3+}/{\mathrm{Fe}}^{2+}$ ratios and are expected for cobalt ferrite films with increasing Co content due to the substitution of Fe${}^{2+}$ by Co${}^{2+}$ [44].

## 4. Conclusions

In summary, the growth behavior and evolving strain of ${\mathrm{Co}}_{x}{\mathrm{Fe}}_{3-x}O_{4}$ single thin films with stoichiometries *x* = 0.6–1.2 grown on MgO(001) were monitored with operando time-resolved specular XRD measurements, which were analyzed using full kinematic diffraction theory. For each film, highly crystalline ordering is observed throughout the entire film growth. However, up to a film thickness of $d_{\mathrm{film}}$∼12 nm, all ${\mathrm{Co}}_{x}{\mathrm{Fe}}_{3-x}O_{4}$ films exhibited enhanced vertical compressive strain attributed to the formation of APBs during the growth of the Co ferrite films on MgO(001). The strain, however, partly releases with increasing film thickness. The residual constant vertical strain for film thicknesses above 12 nm is reconcilable with the model of pseudomorphic growth on MgO substrates. Furthermore, the vertical-layer distance of the ${\mathrm{Co}}_{x}{\mathrm{Fe}}_{3-x}O_{4}$ films increases with increasing Co content, while all films exhibit overall very small surface roughnesses. Nevertheless, LEED measurements point to a slight increase in the surface defect density with increasing Co content. In contrast to this, the roughness of the substrate–film interface decreases for increasing Co content, as indicated in both the XRR and XRD analyses. HAXPES experiments confirm the underlying stoichiometry of the ${\mathrm{Co}}_{x}{\mathrm{Fe}}_{3-x}O_{4}$ films and reveal a reduced amount of ${\mathrm{Fe}}^{2+}$ cations for higher *x* due to the expected gradual substitution of ${\mathrm{Fe}}^{2+}$ by ${\mathrm{Co}}^{2+}$.

Finally, considering the evolving strain of the different ${\mathrm{Co}}_{x}{\mathrm{Fe}}_{3-x}O_{4}$ films produced, our results may open up new perspectives for strain engineering physical properties of ultrathin ${\mathrm{Co}}_{x}{\mathrm{Fe}}_{3-x}O_{4}$ films depending on the amounts of Co. Accurate knowledge of the strain accumulation in the films as provided here allows specific physical (magnetic or electronic) properties to be targeted to meet or even surpass the criteria required for the films to be used in spintronics. For instance, it has to be explored how the APB-related strain influences the electronic structure, e.g., the spin-dependent band gap and, thus, the use of ultrathin Co ferrite films as spin-filters.

## Figures and Tables

**Table 1:** Structural parameters of the prepared Co${}_{x}$Fe${}_{3-x}$O${}_{4}$ films. The final Co${}_{x}$Fe${}_{3-x}$O${}_{4}$ film thickness $d_{\mathrm{film}}^{\mathrm{XRR}}$ was determined with XRR, while the vertical-layer distance $c_{\mathrm{vert}}$, the film surface roughness $\sigma_{f}^{\mathrm{XRD}}$ and the substrate–film interface roughness $\sigma_{s/f}^{\mathrm{XRD}}$ were determined from the XRD analysis based on kinematic diffraction theory.

Co Content *x*	$d_{\mathrm{film}}^{\mathrm{XRR}}$ (nm)	$c_{\mathrm{vert}}$ (pm)	$\sigma_{f}^{\mathrm{XRD}}$ (pm)	$\sigma_{s/f}^{\mathrm{XRD}}$ (pm)
0.6	28.2 ± 0.2	209.0 ± 0.1	60 ± 20	710 ± 30
0.9	32.1 ± 0.2	209.2 ± 0.1	70 ± 20	670 ± 30
1.2	28.6 ± 0.2	209.5 ± 0.1	70 ± 20	630 ± 30

## Data Availability

The data presented in this study are available on reasonable request from the corresponding author.
